# Supplementary material for: Conditional chemoconnectomics (cCCTomics) as a strategy for efficient and conditional targeting of chemical transmission
Source: eLife. 2024 Apr 30;12:RP91927. doi: 10.7554/eLife.91927 (PMC11060718; doi:10.7554/eLife.91927)
Supplement: Figure 3—source data 3. [file elife-91927-fig3-data3.zip › Figure 3 source data3 part 1.zip!/Figure 3-Source data 3-part1/1071f2-1-CRISPResso_Report_ztwktvfp.zip!/CRISPResso_on_1071f2_3/9.Alleles_frequency_table_around_sgRNA_gcagaaggatttgtaaatgg.pdf]

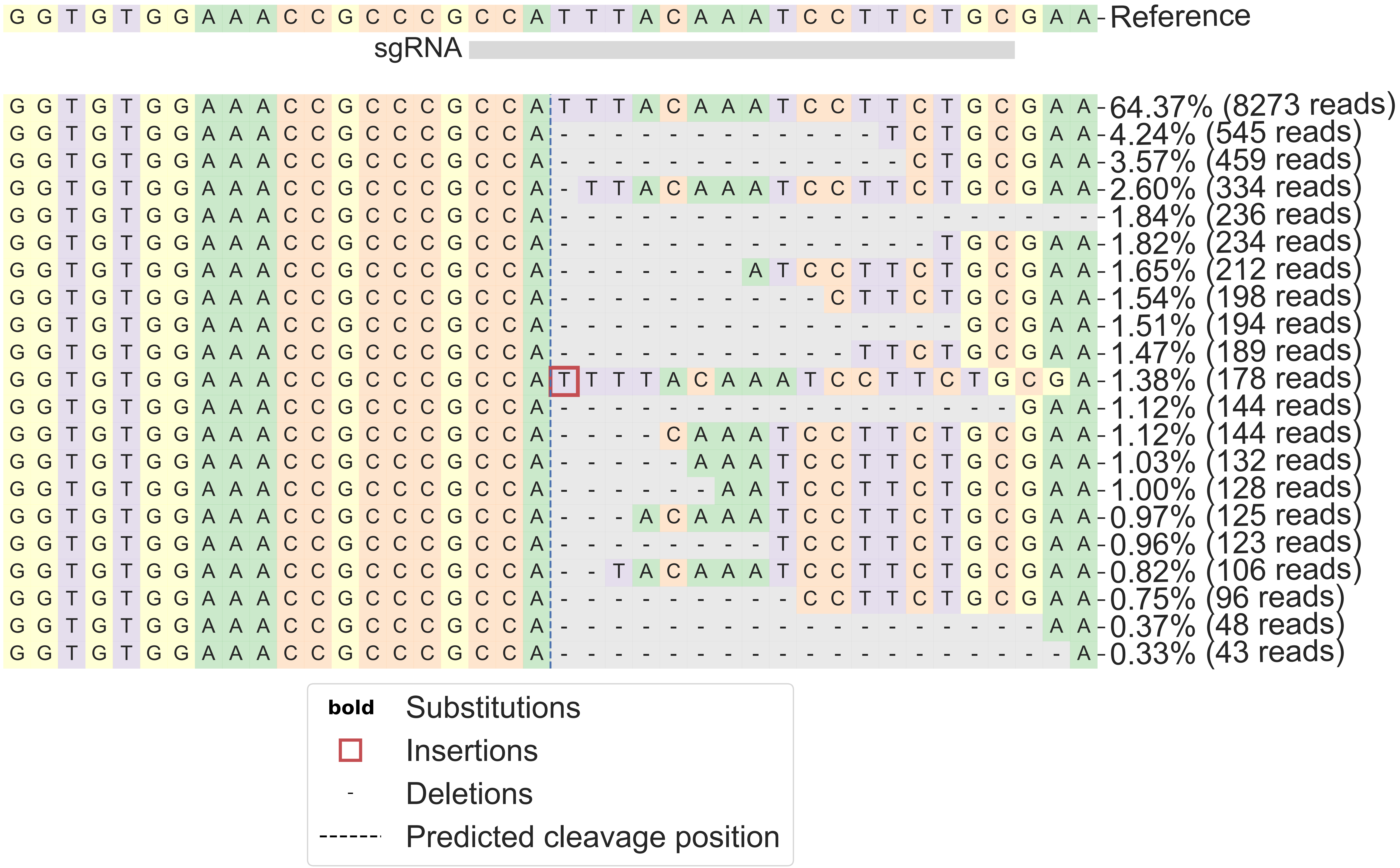
GGTGTGGAAACCGCCCGCCATTTACAAATCCTTCTGCGAA Reference
sgRNA
GGTGTGGAAACCGCCCGCCATTTACAAATCCTTCTGCGAA 64.37% (8273 reads)
GGTGTGGAAACCGCCCGCCA------------TCTGCGAA 4.24% (545 reads)
GGTGTGGAAACCGCCCGCCA-------------CTGCGAA 3.57% (459 reads)
GGTGTGGAAACCGCCCGCCA-TTACAAATCCTTCTGCGAA 2.60% (334 reads)
GGTGTGGAAACCGCCCGCCA-------------------- 1.84% (236 reads)
GGTGTGGAAACCGCCCGCCA--------------TGCGAA 1.82% (234 reads)
GGTGTGGAAACCGCCCGCCA-------ATCCTTCTGCGAA 1.65% (212 reads)
GGTGTGGAAACCGCCCGCCA----------CTTCTGCGAA 1.54% (198 reads)
GGTGTGGAAACCGCCCGCCA---------------GCGAA 1.51% (194 reads)
GGTGTGGAAACCGCCCGCCA-----------TTCTGCGAA 1.47% (189 reads)
GGTGTGGAAACCGCCCGCCATTTTACAAATCCTTCTGCGA 1.38% (178 reads)
GGTGTGGAAACCGCCCGCCA-----------------GAA 1.12% (144 reads)
GGTGTGGAAACCGCCCGCCA----CAAATCCTTCTGCGAA 1.12% (144 reads)
GGTGTGGAAACCGCCCGCCA-----AAATCCTTCTGCGAA 1.03% (132 reads)
GGTGTGGAAACCGCCCGCCA------AATCCTTCTGCGAA 1.00% (128 reads)
GGTGTGGAAACCGCCCGCCA---ACAAATCCTTCTGCGAA 0.97% (125 reads)
GGTGTGGAAACCGCCCGCCA--------TCCTTCTGCGAA 0.96% (123 reads)
GGTGTGGAAACCGCCCGCCA--TACAAATCCTTCTGCGAA 0.82% (106 reads)
GGTGTGGAAACCGCCCGCCA---------CCTTCTGCGAA 0.75% (96 reads)
GGTGTGGAAACCGCCCGCCA------------------AA 0.37% (48 reads)
GGTGTGGAAACCGCCCGCCA-------------------A 0.33% (43 reads)
bold Substitutions
Insertions
Deletions
Predicted cleavage position
